# A Feigned Tumor of the Jaw

**Published:** 1869-10

**Authors:** Macleod Cameron


A Feigned Tumor of the Jaw.—Emamun, a Mussulmani, aged fifteen, was brought to me on November 20th by her parents. They stated that upwards of a year before they had observed a small tumor near the angle of the lower jaw on the left side. It continued to increase slowly; native practitioners failed to give relief; and at last, despairing of a cure, they had brought her to have it removed by operation. There was a tumor on the left side of the face, rounded, of the size of a tea-cup. The skin slid easily over it, and its most prominent part was dusky red, and apparently on the point of ulcerating. The tumor was firm, of a bony consistence, and seemed equally connected with both jaws. The lower jaw was fixed, the mouth nearly closed, and the girl complained of great pain. In spite of the suffering she had undergone, she had not lost flesh, and the right cheek was plump and rounded. On separating the lips, to inspect as far as possible the interior of the mouth, I observed the ends of two flat bands of a dark color which hung from the tumor into the mouth. On inspecting these somewhat minutely (which was a matter of some difficulty, as she was perpetually starting back, and complaining of great pain) I noticed certain lines which seemed to me to indicate either that the bands were pieces of cloth inserted into a cavity in the tumor, or that cloth of some sort had been recently placed in contact with them, so as to leave its impression. I asked the parents if any cloth had been introduced into the mouth; but they asserted that such was not the case, and the girl corroborated their statement. I now seized the bands with forceps, and, using a little force, succeeded in removing it; the girl shrieked loudly, and endeavored to seize my hand. The band was simply a piece of cloth. On examining the mouth I saw what was undoubtedly a second piece of cloth, which I also removed, and thus I went on removing piece after piece till every vestige of the tumor disappeared. The girl looked foolish and sulky. The parents seemed stupified, and could not at once realize that their daughter's illness was pure deception. They brought her to me again on the following day. There was not the slightest trace of disease. The teeth were sound, the jaws well formed. The right cheek was, as I said before, plump and round; the left was thin, and hung flacid and void of expression. The center of the cheek, which formed the most prominent part of the tumor was now shrivelled up, like the skin of a withered apple. The tumor was composed of twenty-three pieces of cloth, weighing, when washed and dried, 4 ounces.—Dr. Macleod Cameron in *Indian Medical Gazettee.*

